# Supplementary material for: Effects of tropical cyclone Jasper at Low Island, northern Great Barrier Reef
Source: Camb Prism Coast Futur. 2025 Nov 11;3:e27. doi: 10.1017/cft.2025.10017 (PMC12722042; doi:10.1017/cft.2025.10017)
Supplement: Muecke et al. supplementary material [file S2754720525100176sup001.pdf]

Supplementary Figures and Tables

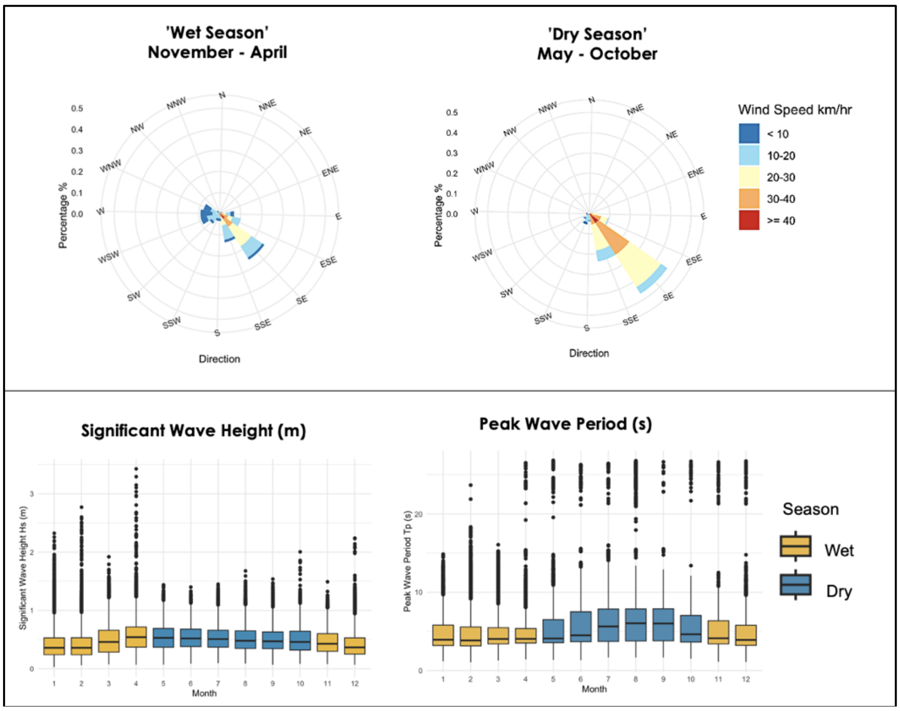

Figure 1) a) Wind speed and direction recorded at Port Douglas. (b) Significant wave height (m) and peak wave period (s) recorded at the Cairns WaveRider buoy. Months 1 - 12 correspond to January - December, respectively.

Table 1 Drone Survey Details

Survey Date	Drone Model	Altitude (m)	Front/Side Overlap (%)	Flight Perspective	Ground Resolution (m)	DEM/Survey Error (m)	Notes
14 June 2023	DJI Mavic-3	60	50	Nadir (-90°)	0.017	0.26	Flight during low tide.
31 January 2024	Phantom 4 Pro RTK	60	80	Nadir (-90°)	0.017	0.32	
June 2024	DJI Mavic-3	60	50	-	0.017	0.32	Oblique imagery; no flight over cay per GBRMPA/QPWS rules.
